# Early experience in a breast and ovarian cancer risk management clinic in Malaysia

**DOI:** 10.1186/1897-4287-10-S2-A58

**Published:** 2012-04-12

**Authors:** NA Taib, YL Woo, SY Yoon, R Kartini, MK Thong, CH Yip, SH Teo

**Affiliations:** 1 Department of Surgery, University Malaya Medical Centre, Malaysia; 2 Department of Obstetrics and Gynaecology, University Malaya Medical Centre, Malaysia; 3 Department of Biomedical Imaging, University Malaya Medical Centre, Malaysia; 4 Department of Paediatrics, University Malaya Medical Centre, Malaysia; 5 UM Cancer Research Institute, Malaysia; 6 Cancer Research Initiatives Foundation, Malaysia

## Background

The discovery of *BRCA1* and *BRCA2* in the mid 1990’s has transformed the management of breast and ovarian cancer families. As part of the **M**ala**y**sian **Br**east **Ca**ncer Genetic Study [MyBrCa], University of Malaya and CARIF have established a genetic counseling, genetic testing and risk management clinic for high-risk breast and ovarian cancer families. We studied the uptake and acceptance of screening and prophylactic surgery amongst women who carry a *BRCA1* or *BRCA2* mutation.

## Methods

Between Jan 03 and Dec 10, a total of 384 patients in the MyBrCa Study had full sequencing and large rearrangement analysis of both *BRCA1* and *BRCA2* genes. Of these, 56 index patients (15%) were found to carry deleterious mutations. All patients and their relatives who carry mutations in these genes were offered follow-up in a dedicated risk management clinic. The clinic is run jointly by a consultant breast surgeon, gynae-oncologist, a genetic counselor and supported by a consultant radiologist.

## Results

Of 56 index patients, 40 (71%) chose to know their genetic test results. In addition, 18 female relatives were identified as carriers. Six had passed away. Thus, 52 female carriers are being followed-up [43 affected, 9 unaffected individuals]. Of these, 25 women or 62.5% [24 previously affected by breast cancer and 1 unaffected BRCA carriers] chose to attend a dedicated risk management clinic at University Malaya Medical Centre. Of these 24 affected women, 4 (17%) already had bilateral mastectomies for bilateral breast cancers, 5 (24%) chose to have risk reducing mastectomy (RRM), the remaining 16 (76%) chose breast surveillance [only 6 had MRI] and none took up chemoprevention with Tamoxifen. Of the 19 women who did not have previous TAHBSOs, 12 (63%) chose risk reducing salpingo-oopherectomy (RRSO).

## Conclusions

The uptake of RRM was lower than RRSO. This study shows that although specialized risk management clinics are acceptable in a high proportion of clients, reasons for not wanting to attend a risk management clinic should be explored.

